# Dynamics of Different Bacterial Communities Are Capable of Generating Sustainable Electricity from Microbial Fuel Cells with Organic Waste

**DOI:** 10.1264/jsme2.ME13140

**Published:** 2014-04-30

**Authors:** Shuji Yamamoto, Kei Suzuki, Yoko Araki, Hiroki Mochihara, Tetsuya Hosokawa, Hiroko Kubota, Yusuke Chiba, Owen Rubaba, Yosuke Tashiro, Hiroyuki Futamata

**Affiliations:** 1 Department of Applied Chemistry and Biological Engineering, Graduate School of Engineering, Shizuoka University, Hamamatsu, Shizuoka 432–8561, Japan

**Keywords:** population dynamics, community structure, microbial fuel cell, *Geobacter*, selective enrichment

## Abstract

The relationship between the bacterial communities in anolyte and anode biofilms and the electrochemical properties of microbial fuel cells (MFCs) was investigated when a complex organic waste-decomposing solution was continuously supplied to MFCs as an electron donor. The current density increased gradually and was maintained at approximately 100 to 150 mA m^−2^. Polarization curve analyses revealed that the maximum power density was 7.4 W m^−3^ with an internal resistance of 110 Ω. Bacterial community structures in the organic waste-decomposing solution and MFCs differed from each other. Clonal analyses targeting 16S rRNA genes indicated that bacterial communities in the biofilms on MFCs developed to specific communities dominated by novel *Geobacter*. Multidimensional scaling analyses based on DGGE profiles revealed that bacterial communities in the organic waste-decomposing solution fluctuated and had no dynamic equilibrium. Bacterial communities on the anolyte in MFCs had a dynamic equilibrium with fluctuations, while those of the biofilm converged to the *Geobacter*-dominated structure. These bacterial community dynamics of MFCs differed from those of control-MFCs under open circuit conditions. These results suggested that bacterial communities in the anolyte and biofilm have a gentle symbiotic system through electron flow, which resulted in the advance of current density from complex organic waste.

Technologies that convert organic waste to energy are needed for the construction of a sustainable society. Renewable organic waste accounted for approximately 60% (3.2×10^8^ tons year^−1^) of the total amount of waste produced in Japan in 2008 (40). Although chemical and biological approaches to sustainable energy production, such as methane, ethanol, and hydrogen, have been developed, many of these approaches have encountered technical and economical hurdles (12, 14). Microbial fuel cells (MFCs) represent an alternative strategy capable of directly converting organic waste to electricity (34, 35).

MFCs are devices that exploit microorganisms as “biocatalysts” to generate electric power from organic matter. MFC systems have been researched as a method of recovering energy from organic waste in the form of electrical power (17, 34, 35, 43) and generating power from aquatic sediments on the bottom of the ocean (37, 53), rice paddy field soil (25, 27), wheat straw biomass (56) or cellulose (45). However, the current densities produced have been too low for practical use.

Many studies have been conducting to improve the potential of MFCs, including the development of a harness, electrode and proton exchange membrane (7, 21, 24, 32, 33, 46) or the analysis of exoelectrogens in pure cultures (15, 57), especially *Geobacter* and *Shewanella* strains (2, 4, 44). Two kinds of extracellular electron transfer (EET) mechanisms have been identified from a bacterial cell to an anode; *Geobacter* spp. generally uses a direct EET mechanism by adhering to electrodes (19, 55), on the other hand, *Shewanella* spp. use indirect EET through mediator compounds (38). Although *Geobacter* strains are known as high electricity-producing bacteria, the electricity-producing activities of complex or mixed cultures were shown to be higher than those of pure *Geobacter* cultures (25). These findings indicated that it may be possible to produce higher current density by controlling the complex microbial ecosystem in the anode chamber. To practically use MFCs, complex organic matter (*i.e.*, food garbage and wastewater) has to be supplied as electron donors, while complex microbial communities have to simultaneously develop in the anode chamber. However, strategies to control microbial ecosystems capable of efficiently producing electricity have yet to be developed. The aim of the present study was to characterize the relationship between bacterial community structures and their electricity- producing properties, and also to evaluate the performance of MFCs continuously supplied with real organic waste.

## Materials and Methods

### MFC configuration and operation

**Fig. 1:**
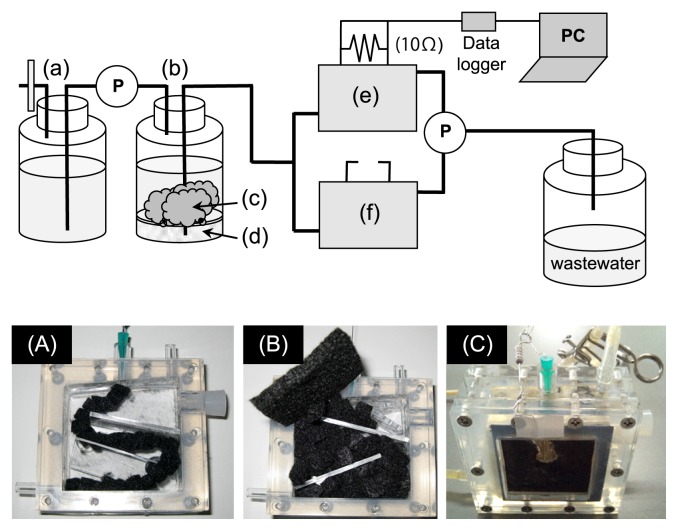
Schematic diagram of the experimental system used in the present study. (a); NaHCO_3_ solution (2.5 g L^−1^, pH 7.5), (b); organic waste-decomposing tank (the tank), (c); organic waste, (d); sea sand, (e); MFC (closed circuit), (f); control MFC (open circuit), P; pump. Panel (A); this photograph shows the inside of the anode chamber in MFCs. Twenty pieces of cubic graphite felt were directly connected with the platinum wire. Panel (B); A total of 115 pieces of cubic graphite felt were set in the anode chamber. Panel (C); This shows the MFCs used in this study.

A mediator-less and air-cathode MFC was used to examine power generation with organic waste, in which paddy field soil was inoculated into the MFC as the initial inoculum. Fig. 1 shows the experimental system used in this study. A carbon paper electroplated with platinum (0.5 mg cm^−2^) on one side was used as the cathode electrode (Chemix, Sagamihara, Japan), providing a total projected cathode surface area (on one side) of 16 cm^2^. A proton exchange membrane (Nafion 117, DuPont, Delaware) was placed between the anode and cathode. A total of 135 pieces of cubic (125 mm^3^) graphite felt (Sohgoh-C, Yokohama, Japan) were packed into the anode chamber (36 mL in capacity) and the total projection surface area of the anode was 0.02025 m^2^. Twenty of these pieces were directly connected to the platinum wires (0.3 mm; AlfaAesar). A total of 0.4 g of paddy field soil was inoculated into the MFC with 20 mM lactate as the initial carbon and energy sources, and the electrodes were then connected with an external resister (10 Ω) 5 days after the inoculation. As a control, an MFC was run under an open circuit condition (control MFC) that was also constructed with the same materials. Organic waste was collected from the canteen and 20 g or 40 g of this was placed directly in a bottle (organic waste-decomposing tank) that contained 1 L of NaHCO_3_ solution (2.5 g L^−1^) for pH control. Sea sand was put on the bottom of the organic waste-decomposing tank (denoted as the tank) as a filter bed and the filtered digested solution was continuously fed into MFCs at a feeding rate of 36 mL d^−1^. The hydraulic residence time was 1.0 d. MFC voltage (*V*) was recorded every 5 min across a 10 Ω resistor (*R*) by a data logger connected to a personal computer.

### Bacterial community analyses

The anolytic culture (1.0 mL) was directly sampled from the anode compartment of MFCs and cells were collected by centrifugation for 5 min at 4°C and 20,000×*g*. Pieces of the anode were collected and maintained at −20°C until DNA extraction. DNA was extracted according to the conventional method described by Futamata *et al.* (20).

#### MDS analyses.

Bacterial community structures were also analyzed by denaturing gradient gel electrophoresis (DGGE) analysis that targeted 16S rRNA genes. The variable region, V3 of bacterial 16S rRNA genes (corresponding to positions 341 to 534 in the *Escherichia coli* sequence) was amplified using the primers P2 and P3 (containing a 40 bp GC clamp [41]) and thermal cycler PC320, as described previously (20). A Dcode DGGE system (Bio-Rad Laboratories, Hercules, CA, USA) was used for electrophoresis as recommended by the manufacturer. A total of 10 μL of the PCR-amplified mixture was subjected to electrophoresis in a 10% (w/v) polyacrylamide gel at 200 V for 3.5 h at 60°C. Gel gradients used for separation, which were applied in parallel to the electrophoresis direction, were 35% to 55%. After electrophoresis, the gel was stained with SYBR Green I (FMC Bioproducts) for 30 min as recommended by the manufacturer.

The intensities of bands in the DGGE gel were measured by Gel Doc XR+ (Bio-Rad Laboratories). Multidimensional scaling (MDS) analysis was performed with these band intensities. Since DGGE analysis does not necessarily completely reproduce the same results, all the intensities and locations of the DGGE bands used in the MDS analysis were compensated for by comparing the intensities and locations of common samples in different DGGE gels. MDS analysis, based on the Bray-Curtis index, was used to analyze the dynamics of the bacterial community structure because this index has been recognized as one of the most useful methods for evaluating differences among populations (8, 13). The following equation was used to calculate the Bray-Curtis index.

$$\delta_{\text{AB}}=(\Sigma |{\text{n}}_{\text{A}}-{\text{n}}_{\text{B}}|)/[\Sigma \;({\text{N}}_{\text{A}}+{\text{N}}_{\text{B}})]\;0\leq \delta_{\text{AB}}\leq 1$$

where δ_AB_ means the dissimilarity index between communities A and B, n_A_ and n_B_ mean the intensities of DGGE bands in clusters of A and B, and N_A_ and N_B_ mean the total intensity of DGGE bands in A and B, respectively (3, 18, 39). MDS analysis and cluster analysis were conducted using the R software program v2.12.1 (The R Project for Statistical Computing: http://www.r-project.org/; University of Tsukuba, Japan: http://cran.md.tsukuba.ac.jp/) (51). Commands used in the R software program v2.12.1 were shown in supplemental datum 1. The 3D graph was constructed using RINEARN Graph 3D v.5.2.0.

#### Clonal analyses.

Bacterial community structures were examined using 16S rRNA clone library analysis. The paddy field soil used as the inoculum was analyzed as the sample at day 0. Cells were collected from the tank on day 34 and day 168. Cells were also collected from the anodic liquid and anode biofilm of MFCs on day 34 and day 168. The DNA fragments of 16S rRNA genes were amplified using the primers 5′-AGAGTTTGATCCTGGCTCAG-3′ (corresponding to *Escherichia coli* 16S rRNA gene positions 8 to 27) [5] and 5′-AAGGAGGTGATCCAGCC-3′ (corresponding to *Escherichia coli* 16S rRNA gene positions 1525 to 1542). Amplification was performed with the thermal cycler PC320 (ASTEC, Osaka, Japan) by using 50 μL of the mixture containing 0.5 U of KOD FX DNA polymerase (Toyobo, Osaka, Japan), buffer solution attached with the PCR kit, each deoxynucleoside triphosphate at a concentration of 400 μM, 15 pmol of each primer, and 50 ng of template DNA. The PCR conditions were 2 min to activate the polymerase at 94°C, then 25 cycles of 1 min at 94°C, 1 min at 53°C, and 1 min at 72°C, and finally 10 min of extension at 72°C. The PCR products were assessed by electrophoresis on a 1.5% (w/v) agarose gel in TAE buffer (48) and stained with GelRed™ (Wako, Osaka, Japan). PCR products were cloned into the vector pTA2 and introduced into competent DH5α cells using the Target Clone™-Plus-kit according to the manufacturer’s recommendations. Clones were isolated by screening for blue/white phenotypes and incubated in TB medium amended with kanamycin (50 mg L^−1^). Plasmid DNA was extracted using a Wizard Minipreps DNA Purification System (Promega, Madison, WI, USA) according to the manufacturer’s directions. DNA was digested with *Eco*RI and electrophoresed, thereby confirming whether an insert was of the expected size.

### Sequencing and phylogenetic analyses

Cloned genes were sequenced with an ABI PRISM BigDye Terminator version 3.1 cycle sequencing kit and analyzed with an ABI PRISM 3100-*Avant* genetic analyzer (Applied Biosystems). Sequence data were complied with the GENETYX-MAC program (GENETYX, Tokyo, Japan). 16S rRNA gene sequence data were analyzed for chimeras with the CHIMERA_CHECK program version 2.7, and compared with those retrieved from Ribosomal Database Project II (10). Sequencing data were compared with those deposited in databases using the BLAST homology search system. The multiple alignments of sequences and calculation of the nucleotide substitution rate by Kimura’s two-parameter model (31) were performed using the CLUSTAL W program (54). Distance matrix trees were constructed by the neighbor-joining method (47), and the topology of the trees was evaluated by bootstrapping with 1,000 resamplings (16).

### Real-time PCR to monitor *Geobacter* strains

Real-time PCR assays were performed on genomic DNA to measure the 16S rRNA gene copy number of *Geobacteraceae*. Template DNAs were prepared as described above. The *Geobacter* spp.-specific primers used for real-time PCR were *Geobacteraceae*- 494f (5′-AGGAAGCACCGGCTAACTCC-3′) (22) and Geo825r (5′-TACCCGCTACACCTAGT-3′) (1). Standard DNA fragments were produced using cloned DNA affiliated with the genus of *Geobacter*. The PCR profile consisted of preheating at 95°C for 10 min, followed by 40 cycles of denaturation at 95°C for 10 s, annealing at 65°C for 5 s, and extension at 72°C for 15 s. The fluorescence signal was detected at 72°C in each cycle, and a melting curve was obtained by heating the product to 95°C and cooling to 40°C. The novel *Geobacter* clade-specific primers used for real-time PCR were designed by aligning the *Geobacter* 16S rRNA sequences obtained from this experiment and GenBank; Novel *Geobacter*-f (5′-GAGGCCTCTGAATATGCTTCTGTA-3′) and Novel *Geobacter*-r (5′-AGCATAACGGGTATTAACCGC-3′) (Fig. S4). Standard DNA fragments were produced using a cloned DNA affiliated with the novel *Geobacter* clade. Real-time PCR was conducted under the same conditions used for *Geobacteraceae*. The reaction was performed using a LightCycler FastStart DNA Master SYBR GREEN I kit (Roche Molecular Biochemicals, Indianapolis, IN, USA) and LightCycler system (Roche Diagnostics, Mannheim, Germany) according to the manufacturer’s instructions. The copy number of amplicons was calculated using LightCycler software version 3.52.

### Electrochemical analyses

Voltage across the external resistor (10 Ω) was automatically monitored every 5 min using a data logger (GL200A, Graphtec, Tokyo, Japan) connected to a personal computer. To evaluate the performance of MFCs, a polarization curve was measured using a potentiostat (HAV-110, HOKUTO DENKO, Tokyo, Japan) at 2 mV min^−1^ of a slope range at an approximate interval. Cell-performance indices (open-circuit voltage [*Voc*], short-circuit current density per volume of the anode harness [*Isc*], maximum power density per volume of the anode harness [*P_max_*], and internal resistance [*R_int_*]) were calculated from the slopes of the polarization curves. In some tests, an Ag/AgCl reference electrode (0.199 V versus standard hydrogen electrode [SHE], HX-R6, Hokuto Denko) was placed into the anode compartments to determine individual electrode potentials. Coulombic efficiency was obtained by calculating the ratio of total recovered coulombs by integrating the current over time to the theoretical amount of coulombs that could be produced from organic waste (see Chemical analysis). Detailed information can be found in a previous study (32).

### Chemical analyses

Liquid samples including small particles were collected from the effluent solution of the tank to measure the redox potential, pH, and COD_cr_ by a colorimetric standard method (5220D. Closed Reflux, Colorimetic Method). The redox potential and pH were measured by the electrode (TPX-999Si, Toko Chemical Lab., Tokyo, Japan). COD_cr_ has primarily been used in MFCs (50) because the amount of COD_cr_ removed by anode microbial metabolism can be used to calculate the number of electrons released from organics (1 g of COD_cr_ was shown to be equivalent to 125 mmol of electrons [42]). These liquid samples were also filtered with a syringe filter (Millipore LG [pore size; 0.2 μm, diameter; 13 mm], EMD Millipore, Billerica, MA, USA) and organic acids were then analyzed by HPLC equipped with a Shodex RSpak KC-811 column (300×8.0 mm) (Showa Denko, Kanagawa, Japan) and UV detector. The column oven was set at 50°C. Samples were eluted with 0.1% H_3_PO_4_ solution at a flow rate of 1.0 mL min^−1^ and elutes were monitored at 210 nm. Formate, pyruvate, lactate, butyrate, and acetate were identified according to the retention time, and the concentration was determined by comparing the peak area with that of its respective standard sample.

### Accession numbers

The nucleotide sequences reported here have been deposited in the DDBJ under accession numbers AB716976 to AB717136, AB720077, AB721108 to AB721264, AB721266 to AB721283, AB724116 to AB724215, AB724352 to AB724383, AB731904 to AB731955, and AB844727 to AB844796.

## Results

### Decomposition of organic waste and performance of MFCs

**Fig. 2:**
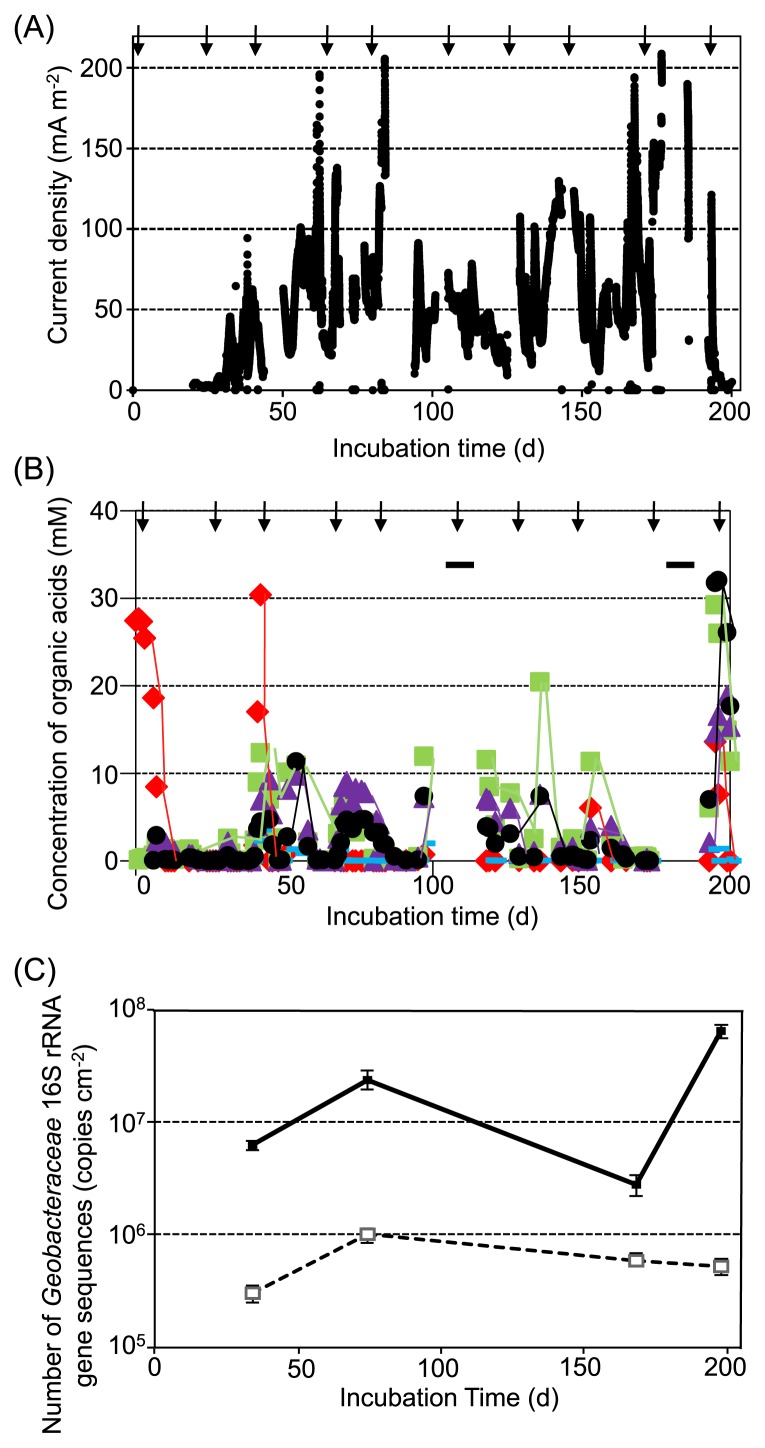
(A) Electricity generation from MFCs with 10 Ω of external resistance. (B) Monitoring of organic acid concentration in the effluence from the organic waste-decomposing tank. Arrows mean the time of addition of organic waste into the organic waste-decomposing tank. Red Diamond: lactate, green square: acetate, black circular: butyrate, purple triangle: propionate, blue bar: formate. Black bars mean the time when organic acid concentrations were not analyzed. (C) Enumeration of *Geobacteraceae* population density by real-time PCR with specific primers. Black square and solid line: the MFC (closed circuit), open circular and broken line: the control-MFC (open circuit).

Organic waste was directly added to the tank [Fig. 1(b)] at every 20 to 40 d interval (Fig. S1) and physicochemical parameters were monitored (Fig. 2, Fig. S2 and S3). Current generation was observed at approximately day 20, and current density then increased to approximately 80 to 120 mA m^−2^ between day 55 and day 85 (Fig. 2A). After the generation of current declined slightly between day 90 and day 125, current-generating activity developed and reached approximately 180 mA m^−2^ between day 165 and day 185 (Fig. 2A). Electrochemical analyses revealed that the maximum current and power densities also increased from 4 mA m^−2^ and 0.12 W m^−3^ to 220 mA m^−2^ and 7.4 W m^−3^, respectively, while *V*oc was stable at 470 to 500 mV (Table 1). Internal resistance significantly decreased from 2300 Ω to 110 Ω.

Lactate, acetate, propionate, formate, and butyrate were detected and their concentrations varied from several mM to 30 mM (Fig. 2B). In the initial stage (between day 0 and day 45), lactate was detected after the addition of organic waste, and the maximum concentration was approximately 30 mM. On the other hand, acetate, propionate, and butyrate were detected after the initial stage, and their concentrations varied from approximately 5 mM to 30 mM (between day 45 and day 200) (Fig. 2B). In the middle stage (between day 95 and day 150), the proportion of acetate detected in organic acids was higher than that in the other stages. The redox potential and pH ranged from −200 mV to −450 mV and from 5.6 to 7.8, respectively (Fig. S2). COD_cr_ and coulombic efficiency ranged from 375 mg L^−1^ to 11,000 mg L^−1^ and from 0.3% to 46%, respectively (Fig. S3).

### Bacterial community structure

**Fig. 3:**
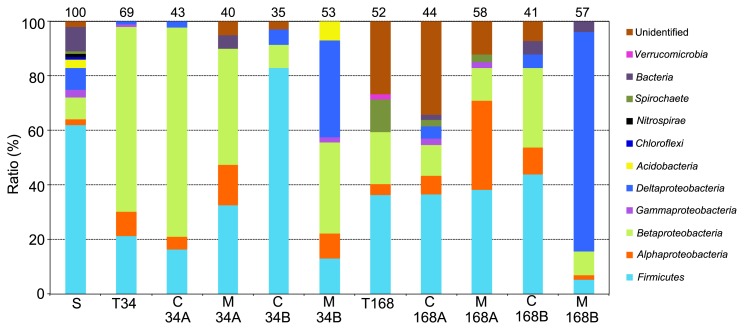
Phylogenetic distribution of 16S rRNA gene clones in soil, the organic waste-decomposing tank (the tank), control-MFCs, and MFCs. S; soil, T34; the tank on day 34, C34A; anolyte in control-MFCs on day 34, M34A; anolyte of MFCs on day 34, C34B; biofilm in control-MFCs on day 34, M34B; biofilm in MFCs on day 34, T168; the tank on day 168, C168A; anolyte in control-MFCs on day 168, M168A; anolyte in MFCs on day 168, C168B; biofilm in control-MFCs on day 168, M168B; biofilm in MFCs on day 168. The number above each bar indicates the total number of sequenced clones.

Clonal analyses targeting the 16S rRNA gene were performed to investigate the bacterial community structure in paddy filed soil, the tank, control-MFCs, and MFCs (Fig. 3). The results of sequence analyses were summarized in Supplemental Table S1. Sequence analysis revealed that *Firmicutes*, α- and β*-proteobacteria* (denoted the Fαβ-group) dominated over 90% of all communities in the tank and anolytes of both the control-MFCs and MFCs on day 34 (Fig. 3. Lanes T34, C34A, M34A). The community structure of soil was more diverse than those of the tank, the control- MFCs and the MFCs in which the proportion of the Fαβ-group to total soil communities was 76% (Fig. 3. Lane S). Although the Fαβ-group also dominated 92% of all biofilm communities in control-MFCs on day 34 (Fig. 3. C34B), it only dominated 55% of those in MFCs (Fig. 3. M34B).

Forty-eight out of the 100 clones analyzed were affiliated with *Acetobacterium* sp. HAAP-1 (98% identity) in *Firmicutes*, which was most frequently detected in paddy field soil. The clones related to *Firmicutes bacterium* BV9-3a (94% identity), belonging to *Firmicutes*, and those related to *Holophage foetida* (89% identity), belonging to *Betaproteobacteria*, were the second and third most frequently detected, respectively (Fig. 3. Lane S). Forty of the 46 clones belonging to β*-proteobacteria* were *Microvirgula aerodenitrificans* (over 99.8% identity), which were most frequently detected in the tank on day 34 (Fig. 3. Lane T34).

These results indicated that the anolytic bacterial community structures in control-MFCs and MFCs were similar to those in the tank on day 34, (Fig. 3. Lane T34, C34A, and M34A). However, their biofilm community structures were different (Fig. 3. Lane T34, C34B, and M34B). On the other hand, differences were observed in bacterial community structures among the tank and anolytes in control-MFCs and MFCs on day 168. For example, the percentage of α*-proteobacteria* in the anolytic bacterial community in MFCs (33%) was significantly higher than those in the tank (1%) and control-MFCs (7%) (Fig. 3. Lanes T168, C168A, and M168A). Furthermore, the biofilm bacterial community structure in MFCs was significantly different from that in control-MFCs (Fig. 3. Lanes C168B and M168B). δ*- proteobacteria* in particular dominated 81% of the biofilm bacterial communities in MFCs on day 168 (Lanes M168B), and only consisted of *Geobacter* spp. (Table S1). On the other hand, δ*-proteobacteria* dominated half of the biofilm bacterial communities in MFCs on day 34 (Fig. 3. Lane M34B). These results indicated that bacterial communities adapted to the respiration process by using anode electrodes as electron acceptors.

### Phylogenetic analysis and enumeration of *Geobacteraceae* populations

Since *Geobacter* species are known to be high electricity- producing bacteria, improvements in MFCs are important for analyzing and monitoring the communities in MFCs. Clones closely related to *Geobacteraceae* were detected in paddy field soil (2 clones), the biofilm in control-MFCs on day 34 (2 clones), the anolyte and biofilm in control-MFCs on day 168 (2 clones and 2 clones, respectively), and the biofilm in MFCs on day 34 and day 168 (17 clones and 46 clones, respectively).

**Fig. 4:**
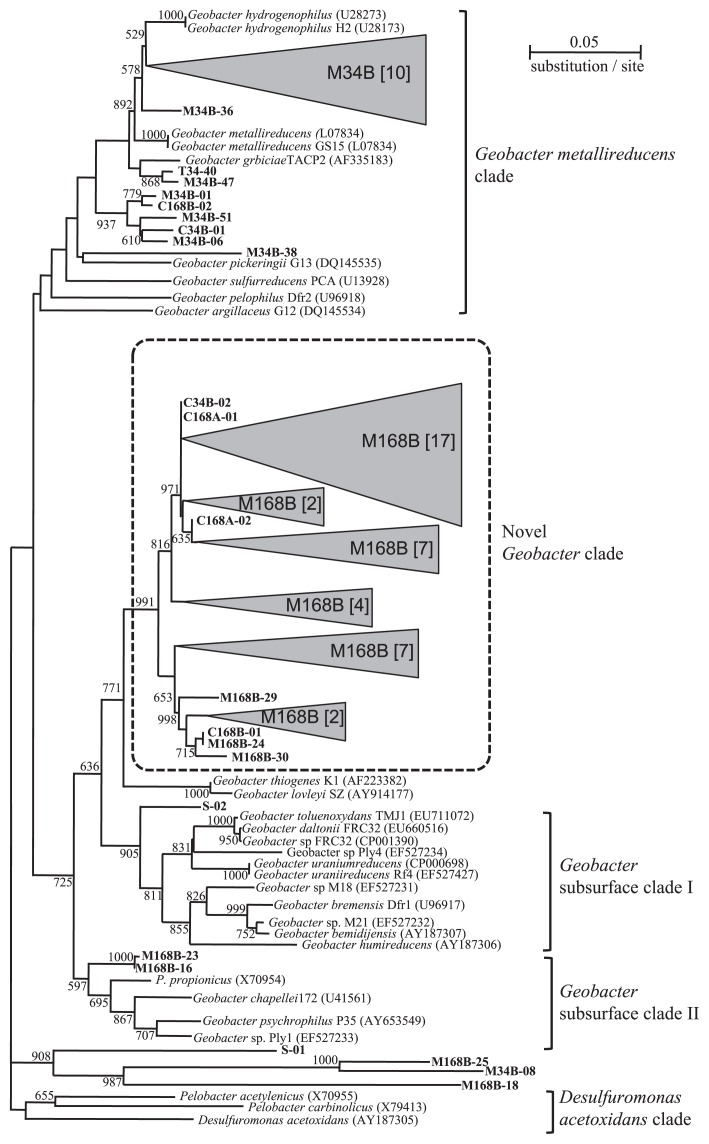
A phylogenetic tree based on partial 16S rRNA gene sequences of representative *Geobacter* isolates and *Geobacteraceae* sequences phylotypes retrieved in this study (indicated by bold letters). The numbers of clones retrieved from different libraries are shown in square brackets. The names for the clone libraries are abbreviated as follows: M34B, biofilm in MFCs on day 34; C34B, biofilm in control-MFCs on day 34; C168B, biofilm in control-MFCs on day 168; C168A, anolyte in control- MFCs on day 168, M168B, biofilm in MFCs on day 168; S, soil. *Desulfuromonas acetoxidans* was used as an outgroup. Bootstrap value (1000 trials, only > 500 are shown) are indicated at branching points. The bar indicates 5% sequence divergence. Accession numbers are shown in parentheses.

Phylogenetic analysis revealed that *Geobacter* communities were grouped into four clusters consisting of the *Geobacter metallireducens* clade, *G.* subsurface clades I, II, and a novel clade (Fig. 4). Most *Geobacter* spp. from the biofilm in MFCs on day 34 belonged to the *G. metallireducens* clade (10 clones out of 16 shared 97% identity with *Geobacter hydrogenophilus*), while those on day 168 belonged to the novel clade (42 clones out of 46 shared 94% identity with *Geobacter lovleyi* SZ). This result indicates that the *Geobacter* community was markedly changed and converged to a specific genotype. Clonal analysis targeting the 16S rRNA gene showed that clones closely related to *Geobacter* spp. dominated the biofilm population, approximately 50% and 80% of all analyzed clones on day 34 and day 168, respectively (Fig. 3).

Real-time PCR analyses revealed that the population density of *Geobacteraceae* in the anode biofilm in MFCs increased from approximately 6.3±0.25×10^6^ copies cm^−2^ on day 34 (current density was 28 mA m^−2^) to 2.4±0.12×10^7^ copies cm^−2^ on day 74 (74 mA m^−2^). Population density decreased to 2.8±0.10×10^6^ copies cm^−2^ on day 168 (164 mA m^−2^), but increased again to 6.4±0.22×10^7^ copies cm^−2^ on day 198 (7.5 mA m^−2^) (Fig. 2). The population density of the novel *Geobacter* clade was approximately 5.3±0.86×10^6^ copies cm^−2^ on day 74 and 1.4±0.05×10^7^ copies cm^−2^ on day 168. On the other hand, population density in the anode biofilm in control-MFCs increased from approximately 3.0±0.12×10^5^ copies cm^−2^ on day 34 to 1.0±0.08×10^6^ copies cm^−2^ on day 74, but decreased to 5.3±0.21×10^5^ copies cm^−2^ on day 198 [Fig. 2(C)].

### Bacterial community dynamics

**Fig. 5:**
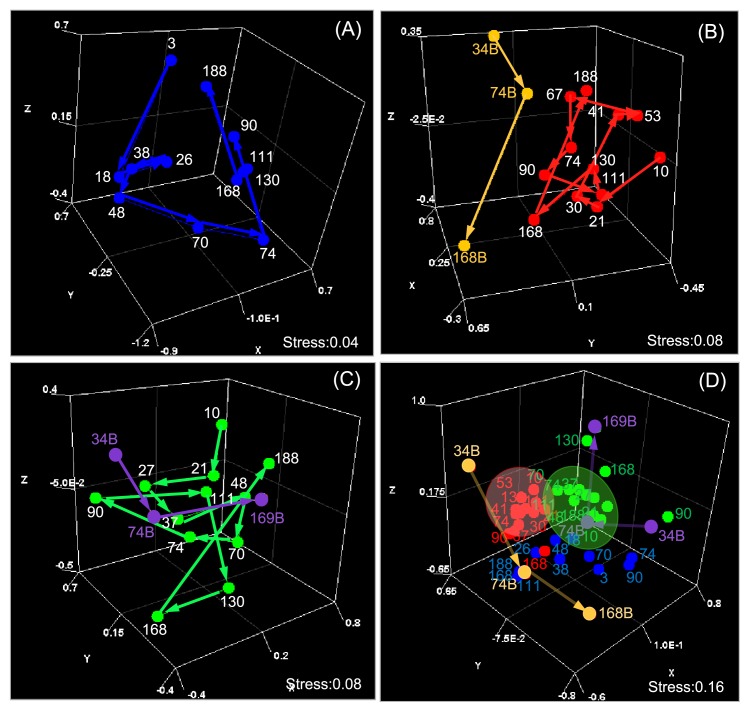
Multidimensional scaling (MDS) analyses based on DGGE profiles. (a); the organic waste-decomposing tank, (b); biofilm (orange) and anolyte (red) in MFCs, (c); biofilm (purple) and anolyte (green) in control-MFCs, (d); All plots were shown under the same scales. The number beside the plots means the sampling date and “B” beside the number means the biofilm sample.

MDS analyses based on DGGE profiles were performed to understand the dynamics of the bacterial community in the MFC system. All stress values were less than 0.20, which indicated that these data were valuable statistically. The bacterial community structure in the tank always changed with no dynamic equilibrium (Fig. 5A). On the other hand, the community structures of anolytic bacteria in both control-MFCs and MFCs had dynamic equilibrium with fluctuations, and also differed from each other (Fig. 5D). The biofilm-community structures in both MFCs did not have dynamic equilibrium and developed different structures from those of the anolytic community structures (Fig. 5B–D). The dissimilarity index value of the tank changed within the scope from 0.22 to 0.96 and the average value was 0.71±0.18. The dissimilarity index values of the anolyte bacterial community in control-MFCs varied from 0.35 to 0.96 and the average was 0.72±0.14, while these values in MFCs were from 0.32 to 0.84 and the average was 0.62±0.14. These results indicated that the anolyte-bacterial community structure in MFCs fluctuated less than that in control-MFCs. The dissimilarity index values of the biofilm bacterial community in control- MFCs varied from 0.81 to 1.00 and the average was 0.90±0.10, while these values in MFCs were from 0.71 to 1.00 and the average was 0.83±0.15. These results indicated that selective pressure on the surface of the anode was stronger in MFCs than in control-MFCs.

## Discussion

Many studies have been conducted on MFCs, and bacterial metabolism was shown to be of significant importance due its effect on the efficiency of electricity production (52). The metabolic processes of microorganisms under anaerobic conditions connect with each other, and this is known as interspecies hydrogen transfer. Bacterial community structures are known to be affected by substrates supplied to the anode of MFCs (6, 28, 49). A clear understanding of the anaerobically complex microbial ecosystem in the anode of FCs is important when attempting to improve MFCs for practical use. The results obtained in the present study revealed the relationship between anolyte and anode biofilm bacterial communities and the electrochemical properties of the MFCs used, and also provided an insight into why the performance of MFCs was increased and maintained during this experiment [Fig. 2(A) and Table 1] regardless of the different components of organic waste supplied as electron donors (Fig. S1).

The MFCs used in this study had a power density of approximately 1.1 W m^−3^ (or 2.0 W m^−2^) with electricity production of 100 mA m^−2^. Although these MFCs were the middle to high performance type (28, 49, 56), the development of MFCs, in which performance is increased 1000-fold, is needed for their practical application. pH was almost stable at approximately 6.5 irrespective of diverse and complex organic waste (Fig. S1 and S2), which contributed to the sustainable production of electricity. On the other hand, no obvious positive correlation was observed between current density and redox potential [Fig. 2(A) and Fig. S2]. There appeared to be a negative correlation between COD and coulombic efficiency (Fig. S3). However, since the current density was almost stable during this experiment, it was suggested that the change of coulombic efficiency was pretense.

Although bacterial communities in the tank had been always fed into control-MFCs and MFCs, MDS analyses demonstrated that the dynamics of bacterial community structures differed between the tank, control-MFCs, and MFCs (Fig. 3 and Fig. 5D). The anolytic bacterial community structure in MFCs fluctuated less than that in control-MFCs, and the dynamic equilibrium of anolytic bacterial community structures in both MFCs differed from each other (Fig. 5D). Furthermore, the difference observed in the biofilm bacterial community structure between control-MFCs and MFCs was larger than that in anolytic bacterial community structures (Fig. 5D). These results demonstrated that the anode electrode as an electron acceptor was direct involved in the dynamics of biofilm bacterial communities on the anode and also indirectly influenced the dynamics of anolytic bacterial communities. Our results suggested that the relationship between the anolytic and biofilm bacterial communities has a gentle symbiotic system through the electron flow. Furthermore, the anaerobic microbial ecosystem may also be controlled by using the anode electrode as an (terminal) electron acceptor. A few studies have also suggested the presence of electric symbiosis between fermenters in an anolyte and exoelectrogens on an anode surface (26, 30, 36).

By combining the results of clonal and MDS analyses, we showed that the dynamics of the biofilm bacterial community in MFCs converged on the selective enrichment of *Geobacter* (Fig. 3 and Fig. 5D). *Geobacteraceae* has been classified into three clades: the *Geobacter metallireducens* clade, and subsurface clades 1 and 2 (23). The key factors for the selective enrichment of *Geobacter* are diverse. Kato *et al.* reported that diverse *Geobacter* spp. were enriched in the soil layer of a batch culture supplied with (semi)conductive iron-oxide species as the terminal electron acceptor and sodium acetate as the electron donor (29). Other studies demonstrated that the addition of acetate stimulated the growth of *Geobacter* in field and pure culture (*Geobacter sulfurreducens* PCA) experiments, resulting in high electroactivity (23, 52). However, a positive correlation was not necessarily observed between the population density of *Geobacter* and acetate concentration in the tank in the present study (Fig. 2). A previous study demonstrated that complex organic acids alleviated the metabolic constraints derived from assimilation or dissimilation (52). Furthermore, lactate, butyrate, and propionate produced in the tank were typically difficult to use as carbon and energy sources, but were identified as electron donors for *Geobacter* (9). Therefore, these complex organic acids may have stimulated the growth and electroactivity of *Geobacter* in MFCs. These processes enabled the enrichment of *Geobacter* on the anode surface, resulting in an increase in the production of electricity. Phylogenetic analysis showed that the unique phylotype of *Geobacter*, which shared 94% identity with *Geobacter lovleyi* SZ, was enriched in the biofilm in MFCs on day 168 (Fig. 4). Since real-time PCR results demonstrated that the population of the novel *Geobacter* clade was 5-fold higher than that of *Geobacteraceae* detected with previous primer sets (*Geobacteraceae*-494f/Geo825r) on day 169, the specific primer set for the novel *Geobacter* clade designed here was useful for understanding the dynamics of *Geobacteraceae*. Commault *et al.* reported that closely related strains of *Geobacter* have different electron transfer capabilities (11), which suggested that the specific *Geobacter* species adapted to complex organic acids and was enriched on the anode in the present study. By combining the results of electrochemical properties (Table 1), we propose that the enrichment of the novel *Geobacter* clade on the anode may have contributed to the establishment of efficient electron transfer from cells to the anode, resulting in the production of a high current density.

## Conclusion

In conclusion, although it has been suggested that the smooth flow of electrons from the electron donor to the electrode through microorganisms is important for efficient electricity production, information regarding the dynamics of the microbial ecosystem in the anode of MFCs supplied continuously with complex substrates remains sparse. This study characterized electron flow in the anode chamber standing on the sight of microbial ecosystem and the results presented herein demonstrated that anolytic and biofilm bacterial communities developed interdependency. *Geobacter* was enriched selectively and naturally on the surface of the anode. It is inherently important to establish how to control the microbial ecosystem. These subjects are currently under investigation in our laboratory.

## Supplementary Information



## Figures and Tables

**Table 1:** Electricity-producing properties of MFCs used in this study

Incubation time (d)	*V*oc (mV)	Maximum current density (mA m^−2^ anode)	Maximum power density (W m^−3^)	Internal resistance (Ω)
27	160	4	0.12	2300
34	500	44	4.9	370
69	300	140	5.3	160
105	475	63	6.7	200
167	470	200	6.9	140
182	500	220	7.4	110
