# Supplementary material for: Dynamics of Different Bacterial Communities Are Capable of Generating Sustainable Electricity from Microbial Fuel Cells with Organic Waste
Source: Microbes Environ. 2014 Apr 30;29(2):145–53. doi: 10.1264/jsme2.ME13140 (PMC4103520; doi:10.1264/jsme2.ME13140)
Supplement: Supplementary file 1 [file 29_145_s1.pdf]

Supplemental datum 1

The program of MDS analysis on R used in this study

```
>library(mvpart)
>read.table("File name.txt")
>x<-read.table("File name.txt")
>gdist(x,method="bray")
>y<-gdist(x,method="bray")
>cmdscale(y,k=3,eig=T)

>library(MASS)
>x<-read.table("File name.txt")
> y<-gdist(x,method="bray")
>mds<-isoMDS(y,k=3)
```

The program shown here is one of some programs for MDS analysis. "File name.txt" is a matrix data which consists of intensities and locations of DGGE bands.

Supplemental figure 1 (Fig. S1)

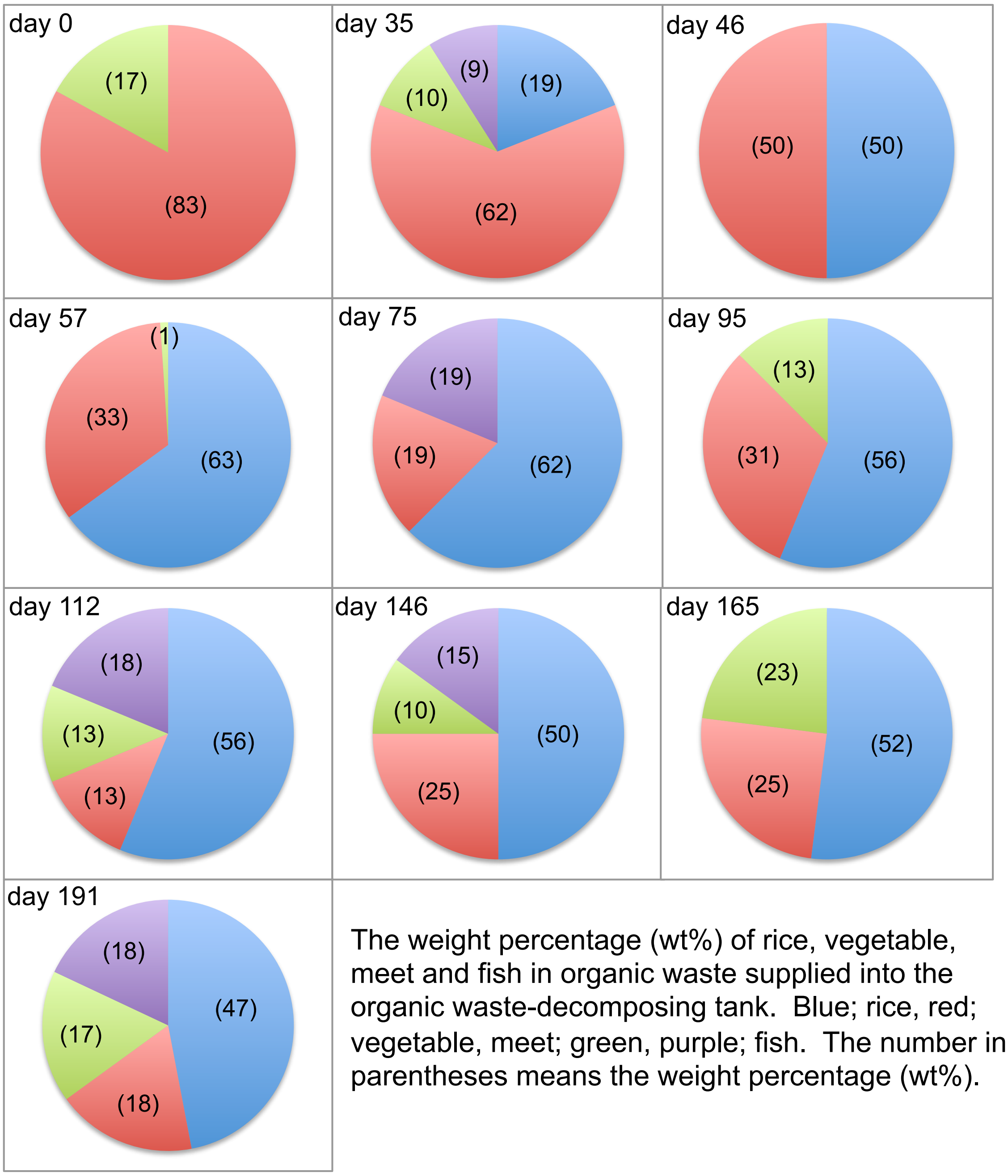

The weight percentage (wt%) of rice, vegetable, meet and fish in organic waste supplied into the organic waste-decomposing tank. Blue; rice, red; vegetable, meet; green, purple; fish. The number in parentheses means the weight percentage (wt%).

Supplemental figure 2 (Fig. S2)

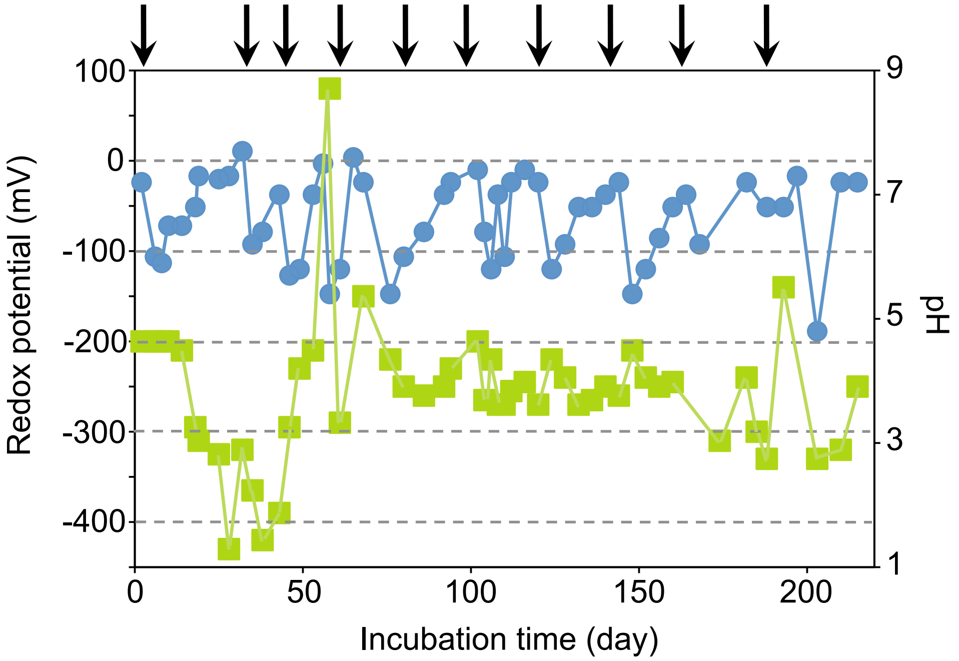

Monitoring of redox potential and pH in the organic waste-decomposing tank. Green square; redox potential, blue circle: pH. Arrows mean the time of addition of organic waste into the organic waste-decomposing tank.

Supplemental figure 3 (Fig. S3)

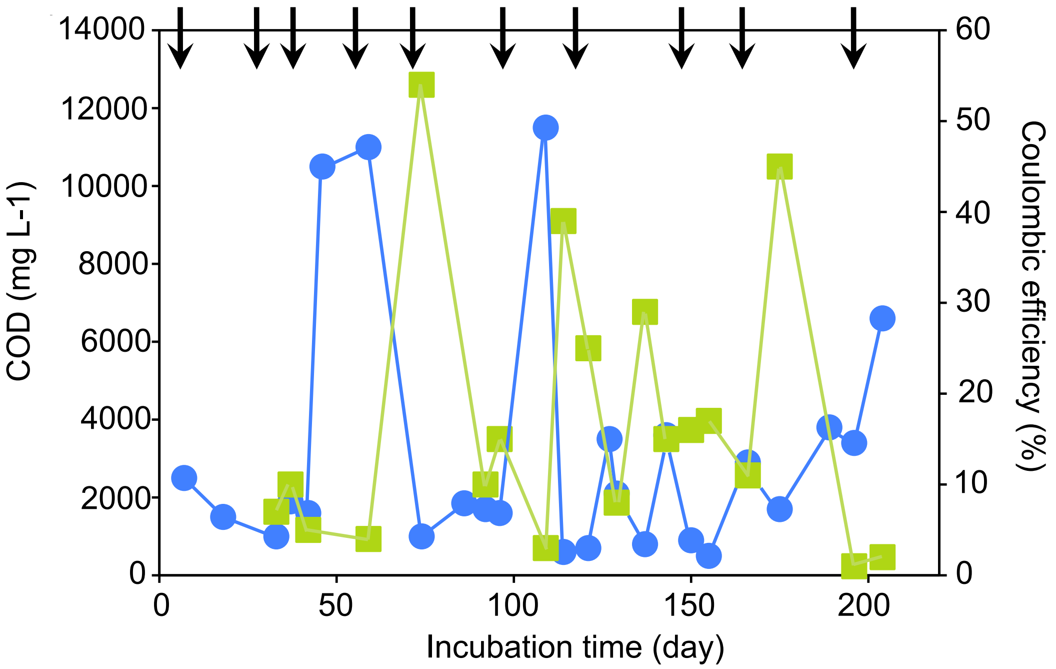

Monitoring of COD in organic waste-decomposing tank and the coulombic efficiency of MFC. Blue circule: COD, green square; coulombic efficiency. Arrows mean the time of addition of organic waste into the organic waste-decomposing tank.

Supplemental figure 4 (Fig. S4)

	188 211	449 469
Geobacter lovley SZ	GGGGCCTCTGAATATGCTCCTGAT	TCGGTTAATACCCGGTTTTCT
M168B-01	GAGGCCTCTGAATATGCTTCTGT**A**	**G**CGGTTAATACCCGTTATGCT
M168B-34	GAGGCCTCTGAATATGCTTCTGT**A**	**G**CGGTTAATACCCGTTATGCT
M168B-17	GAGGCCTCTGAATATGCTTCTGT**A**	**G**CGGTTAATACCCGTTATGCT
M168B-21	GAGGCCTCTGAATATGCTTCTGT**A**	**G**CGGTTAATACCCGTTATGCT
M168B-46	GAGGCCTCTGAATATGCTTCTGT**A**	**G**CGGTTAATACCCGTTATGCT
M168B-08	GAGGCCTCTGAATATGCTTCTGT**A**	**G**CGGTTAATACCCGTTATGCT
M168B-29	GAGGCCTCTGAATATGCTTCTGT**A**	**G**CGGTTAATACCCGTTATGCT
M168B-24	GAGGCCTCTGAATATGCTTCTGT**A**	**G**CGGTTAATACCCGTTATGCT
M168B-30	GAGGCCTCTGAATATGCTTCTGT**A**	**G**CGGTTAATACCCGTTATGCT
	→ Novel *Geobacter*-f	← Novel *Geobacter*-r

Comparison of 16S rRNA sequences of the novel *Geobacter* clade and most closest bacterium (G. lovley SZ). These regions were designed for a set of primers for detection of the novel *Geobacter* clade. Although all sequences shown in figure 4 were compared for design the specific primers, representative sequences are shown here. Numbers above the sequence correspond to the numbering in the 16S rRNA sequence of *G. lovley* SZ.

Paddy field soil (S)		
Mostly related microorganism	Number of clone	Phylogenetic phylum
Acetobacterium	48	*Firmicutes, Clostridia*
Firmicutes bacterium BV9-3a	7	*Firmicutes*
Bacterium ELLin6095	5	*Betaproteobacteria*
Holophaga foetida	4	*Bacteira, Acidobacteria*
Actinomycetales	2	*Bacteria, Actinobacteria*
Bacterium ELLin6067	2	*Betaproteobacteria*
Caulobacter	2	*Alphaproteobacteria*
Desulfobacterium	2	*Deltaproteobacteria*
Desulfotalea psychrophila LSv54	2	*Deltaproteobacteria*
Geobacter	2	*Deltaproteobacteria*
Moorella	2	*Firmicutes, Clostridia*
Psychrosinus fermentans	2	*Firmicutes*
Acidobacterium	1	*Acidobacteria*
Acidobactenaceae bacterium	1	*Acidobacteria*
Actinobacterium TC4	1	*Acidobacteria*
Anaeroarcus burkinensis	1	*Firmicutes*
Anaerobic bacterium MO-CFX1	1	*Bacteria*
Aquicella siphonis	1	*Gammaproteobacteria*
Bacterium TG161	1	*Bacteria*
Caldilinea tarbellica	1	*Chloroflexi*
Candidatus Accumulibacter	1	*Unidentified*
Candidatus Magnetobacterium	1	*Nitrospirae*
Candidatus Magnetoovunm mohavensis	1	*Unidentified*
Desulfosarcina sp. SD1	1	*Deltaproteobacteria*
Luteimonas sp. R-37032	1	*Gammaproteobacteria*
Oscillospiraceae bacterium NML	1	*Firmicutes, Clostridia*
Pseudomonas	1	*Gammaproteobacteria*
Spirochaeta caldaria	1	*Spirochaetes*
Syntrophus sp.	1	*Deltaproteobacteria*
Thermanaeromonas toyohensis	1	*Firmicutes, Clostridia*
Thermovirga lieni	1	*Synergistetes*
Variovorax	1	*Betaproteobacteria*
Total clone number	100	

Organic waste-decomposing tank at day 34 (T34)		
Mostly related microorganism	Number of clone	Phylogenetic phylum
Microvirgula aerodenitrificans	40	*Betaproteobacteria*
Achromobacter sp. BR3	1	*Betaproteobacteria*
Alphaproteobacteirum LJY2	1	*Alphaproteobacteria*
Bosea sp. RNE 2	1	*Alphaproteobacteria*
Brevundimonas sp.	3	*Alphaproteobacteria*
Burkholderiaceae bacterium	1	*Betaproteobacteria*
Clostridium propionicum	1	*Firmicutes, Clostridia*
Delftia sp. S17	1	*Betaproteobacteria*
Delftia tsuruhatensis	1	*Betaproteobacteria*
Escherichia coli	1	*Gammaproteobacteria*
Eubacterium xylanophilum	1	*Firmicutes; Clostridia*
Geobacter hydrogenophilus	1	*Deltaproteobacteria*
Pelosinus sp. BXM	1	*Firmicutes; Negativicutes*
Pelosinus sp. *SH1-2*	2	*Firmicutes; Negativicutes*
Propionispora hippei	2	*Firmicutes; Negativicutes*

Pseudorhodoferax soli	1	*Betaproteobacteria*
Rhodovarius lipocyclicus	1	*Alphaproteobacteria*
Selenomonas lacticifex	1	*Firmicutes; Negativicutes*
Selenomonas ruminantium	1	*Firmicutes; Negativicutes*
Selenomonas sp. oral taxon 442	1	*Firmicutes; Negativicutes*
Sporomusa paucivorans	1	*Firmicutes; Negativicutes*
Sporotalea propionica	2	*Firmicutes; Negativicutes*
Variovorax sp. DC2a-29	1	*Betaproteobacteria*
Veillonellaceae bacterium FCF9B	1	*Firmicutes; Negativicutes*
Veillonellaceae bacterium WK011	1	*Firmicutes; Negativicutes*
Total clone number	69	

Anolytic bacterial communities in the control-MFC at day 34 (C34A)		
Mostly related microorganism	Number of clone	Phylogenetic phylum
Variovorax sp. DC2a-20	8	*Betaproteobacteria*
Zoogloea sp. A5	7	*Betaproteobacteria*
Acetobacterium sp. HAAP-1	3	*Firmicutes, Clostridia*
Acidovorax delafieldii strain 179	3	*Betaproteobacteria*
Microvirgula aerodenitrificans	3	*Betaproteobacteria*
Pelomonas soli	3	*Betaproteobacteria*
Acidovorax sp. GKS12b	2	*Betaproteobacteria*
Pelomonas soli strain SYB4	2	*Betaproteobacteria*
Acidovorax sp. KSP2	1	*Betaproteobacteria*
Bosea sp. JPB-5.11a	1	*Alphaproteobacteria*
Dechlorosoma sp. PCC	1	*Betaproteobacteria*
Lactococcus fujiensis	1	*Firmicutes, Bacillus*
Myxococcus virescens	1	*Deltaproteobacteria*
Acidvorax sp. BSB421	1	*Betaproteobacteria*
Alphaproteobacterium LHY2	1	*Alphaproteobacteria*
Comamonadaceae	1	*Betaproteobacteria*
Firmicutes bacterium BV9-3a	2	*Firmicutes, Clostridia*
Oscillospiraceae bacterium NML 061048	1	*Firmicutes, Clostridia*
Rhodocyclaceae bacterium C-G-TSA7	1	*Betaproteobacteria*
Total clone number	43	

Anolytic bacterial communities in the MFC at day 34 (M34A)		
Mostly related microorganism	Number of clone	Phylogenetic phylum
Microvirgula aerodenitrificans	12	*Betaproteobacteria*
Lactococcus fujiensis	6	*Firmicutes, Bacillus*
Burkholdenaceae bacterium KVD-1700-12	2	*Betaproteobacteria*
Geothrix fermentans	2	*Acidobacteria*
Leuconostoc mesenteroides	2	*Firmicutes, Bacillus*
Pelosinus sp. SHI-2	2	*Firmicutes; Negativicutes*
Rhodocyclaceae bacterium C-G-TSA7	2	*Betaproteobacteria*
Acetobacter ghanensis strain 430A	1	*Alphaproteobacteria*
Acetobacter pasteunaus	1	*Alphaproteobacteria*
Azospirillum sp. B510	1	*Alphaproteobacteria*
Azospirillum sp. YM195	1	*Alphaproteobacteria*
Bacterium str 51885	1	*Unknown bacteria*
Clostridiium puniceum	1	*Firmicutes, Clostidia*
Clostridium acetobutylicum	1	*Firmicutes, Clostidia*
Gluconacetobacter liquefaciens strain BW1	1	*Alphaproteobacteria*
Lactococcus sp. YM05001	1	*Firmicutes, Bacillus*
mercury-resistant bacterium mCFU581	1	*Unknown bacteria*
Sphingomonas sp. CGK-K1	1	*Alphaproteobacteria*
Variovorax sp. DC2a-29	1	*Betaproteobacteria*
Total clone number	40	

Biofilm community structure of the control-MFC at day 34 (C34B)

Mostly related microorganism	Number of clone	Phylogenetic phylum
Veillonellaceae bacterium WK011	11	*Firmicutes, Negativicutes*
Acetobacterium sp. HAAP-1	5	*Firmicutes, Clostridia*
Clostridium puniceum	4	*Firmicutes, Clostridia*
Clostridium sp. Usu-S-R3	2	*Firmicutes, Clostridia*
Selenomonas sp. WG	2	*Firmicutes, Negativicutes*
Acidaminococcus fermentans DSM20731	1	*Firmicutes, Negativicutes*
Bacillus shackietonii	1	*Firmicutes, Bacillus*
Bacterium DCE25	1	*unidentified bacteria*
Clostridium sp. LTR1	1	*Firmicutes, Clostridia*
Geobacter grbiciae	1	*Deltaproteobacteria*
Geobacter lovleyi SZ	1	*Deltaproteobacteria*
Mercury-resistant bacterium mCFU 581	1	*unidentified bacteria*
Microvirgula aerodenitrificans strain LMG432[illegible]	1	*Betaproteobacteria*
Rhodocyclaceae bacterium C-G-TSA7	1	*Betaproteobacteria*
Sporotalea propionica	1	*Firmicutes, Negativicutes*
Zoogloea sp. A5	1	*Betaproteobacteria*
Total clone number	35	

Biofilm community structure of the MFC at day 34 (M34B)

Mostly related microorganism	Number of clone	Phylogenetic phylum
Geobacter hydrogenophilus	7	*Deltaproteobacteria*
Acidovorax facilis	1	*Betaproteobacteria*
Azospirillum brasilense	2	*Alphaproteobacteria*
Brevundimonas sp. AbaT-2	1	*Alphaproteobacteria*
Burkholderiaceae bacterium KVD-1700-12	1	*Betaproteobacteria*
Geobacter grbicium	1	*Deltaproteobacteria*
Geobacter grbiciae	2	*Deltaproteobacteria*
Geobacter hydrogenophilus	6	*Deltaproteobacteria*
Geobacter metallireducens GS-15	1	*Deltaproteobacteria*
Geothrix fermentans	6	*Deltaproteobacteria*
Clostridium sp. LTR1	1	*Firmicutes*
Clostridium mesophilum strain SW408	2	*Firmicutes*
Clostridium sp. PPf35E6	1	*Firmicutes*
Comamonadaceae bacterium OTSz_M_221	1	*Betaproteobacteria*
Cupriavidus sp. JB1B4	1	*Betaproteobacteria*
Delftia sp. BN-SKY3	1	*Betaproteobacteria*
Delftia sp. Cs1-4	5	*Betaproteobacteria*
Delftia sp. SM-1	1	*Betaproteobacteria*
Herbaspirillum sp. JS5-2	1	*Betaproteobacteria*
Microvirgula aerodenitificans	1	*Betaproteobacteria*
Propionispora hippei	1	*Firmicutes*
Pseudorhodoferax soli	1	*Betaproteobacteria*
Rhodocyclus sp. HOD 5	2	*Betaproteobacteria*
Selenomonas lacticifex	1	*Firmicutes*
Sinorihizobium sp. TBB-10-II	1	*Alphaproteobacteria*
Sporotalea propionica	1	*Firmicutes*
Stenotrophomonas maltophilia	1	*Gammaproteobacteria*
Rhizobium sp. OS-B19	1	*Alphaproteobacteria*
Variovorax sp. DC2a-29	1	*Betaproteobacteria*
Total clone number	53	

Organic waste-decomposing tank at day 168 (T168)

Mostly related microorganism	Number of clone	Phylogenetic phylum

Mercury-resistant bacterium mCFU 581	13	unidentified bacteria
Treponema sp. 9:A:D01	6	*Spirochaetes*
Veillonellaceae bacterium WK011	6	*Firmicutes; Negativicutes*
Acidaminococcus fermentans DSM 20731	3	*Firmicutes; Negativicutes*
Megasphaera sueciensis	3	*Firmicutes; Negativicutes*
Ottowia sp. RB1-10B	3	*Betaproteobacteria*
Anaeroarcus burkinensis	2	*Firmicutes; Negativicutes*
Anaerofilum agile	2	*Firmicutes; Clostridia*
Delftia tsuruhatensis	2	*Betaproteobacteria*
Pseudoxanthobacter sp. DDT-1	2	*Alphaproteobacteria*
Aquaspirillum putridiconchylium	1	*Betaproteobacteria*
Aquatic bacterium R1-B22	1	*Bacteria*
beta proteobacterium SAK19	1	*Betaproteobacteria*
Clostridium aminophilum	1	*Firmicutes; Clostridia*
Comamonadaceae bacterium RB3-7	1	*Betaproteobacteria*
Laribacter hongkongensis	1	*Betaproteobacteria*
Lentimonas marisflavi	1	*Verrucomicrobia*
Oscillospiraceae bacterium NML 061048	1	*Firmicutes; Clostridia*
Propionivibrio dicarboxylicus	1	*Betaproteobacteria*
Selenomonas lacticifex	1	*Firmicutes; Negativicutes*
Total clone number	52	

Anolytic bacterial communities in the control-MFC at day 168 (C168A)		
Mostly related microorganism	Number of clone	Phylogenetic phylum
Mercury-resistant bacterium mCFU 581	12	unidentified bacteria
Acidaminococcus fermentans DSM20731	4	*Firmicutes, Negativicutes*
Veillonellaceae bacterium WK011	4	*Firmicutes, Negativicutes*
Mercury-resistant bacterium mCFU	3	unidentified bacteria
Xanthobacter agilis	3	*Alphaproteobacteria*
Megasphaera sueciensis	2	*Firmicutes, Negativicutes*
Acidaminococcus fermentans	1	*Firmicutes, Negativicutes*
Actinomyces sp. 402670/2010	1	*Actinobacteria*
Anaeroarcus burkinensis	1	*Firmicutes, Negativicutes*
Aquaspirillum putndiconchylium	1	*Betaproteobacteria*
betaproteobacterium SAK19	1	*Betaproteobacteria*
Christensenella minuta	1	*Firmicutes, Clostridia*
Delftia acidovorans	1	*Betaproteobacteria*
Geobacter grbiciae	1	*Deltaproteobacteria*
Geobacter lovleyi SZ	1	*Deltaproteobacteria*
Megasphaera paucivorans	1	*Firmicutes, Negativicutes*
Pelomonas soli	1	*Betaproteobacteria*
Rhodocyclaceae bacterium C-G-TSA7	1	*Betaproteobacteria*
Selenomonas lacticifex	1	*Firmicutes, Negativicutes*
Selenomonas sp. WG	1	*Firmicutes, Negativicutes*
Treponema sp. 9 A D01	1	*Spirochaetes*
Xanthomonas sp. shui13-10	1	*gammaproteobacteria*
Total clone number	44	

Anolytic bacterial communities in the MFC at day 168 (M168A)		
Mostly related microorganism	Number of clone	Phylogenetic phylum
Xanthobacter agilis	13	*Alphaproteobacteria*
Acidaminococcus fermentans DSM20731	11	*Firmicutes, Negativicutes*
Mercury-resistant bacterium mCFU581	5	*Unknown bacteria*
Bosea sp. 7F	3	*Alphaproteobacteria*
Delftia tsuruhatensis	3	*Betaproteobacteria*
Megasphaera sueciensis	3	*Firmicutes,*
Anaeroarcus burkinensis	2	*Firmicutes, Negativicutes*

Devosia riboflavina	2	*Alphaproteobacteria*
Achromobacter	1	*Betaproteobacteria*
Acidaminococcus sp. DJF-RP55	1	*Firmicutes, Negativicutes*
Anaerofilum agile	1	*Firmicutes, Clostidia*
Brevundimonas sp. H208	1	*Alphaproteobacteria*
Christensenella minuta	1	*Firmicutes,*
Delftia acidovorans	1	*Betaproteobacteria*
Delftia sp. R-41392	1	*Betaproteobacteria*
freshwater bacterium LH6-8	1	*Bacteria*
Lactobacillales bacterium HY-36-1	1	*Firmicutes,*
Oscillospiraceae bacterium NML 061048	1	*Firmicutes, Clostidia*
Pseudomonas aeruginosa	1	*gammaproteobacteria*
Selenomonas lacticifex	1	*Firmicutes; Negativicutes*
Thauera mechemichensis	1	*Unknown bacteria*
Treponema brennaborense DSM12168	1	*Spirochaetes*
Treponema porcinum	1	*Spirochaetes*
Zoogloea resiniphila	1	*Betaproteobacteria*
Total clone number	58	

Biofilm community structure of the control-MFC at day 168 (C168B)

Mostly related microorganism	Number of clone	Phylogenetic phylum
Ottowia sp. RB1-10B	10	*Betaproteobacteria*
Veillonellaceae bacterium WK011	6	*Firmicutes, Negativicutes*
Clostridium butyricum	3	*Firmicutes, Clostridia*
Megasphaera paucivorans	3	*Firmicutes, Negativicutes*
Mercury-resistant bacterium mCFU 581	2	*unidentified bacteria*
Pseudoxanthobacter sp. DDT-1	2	*alphaproteobacteria*
Xanthobacter agilis	2	*alphaproteobacteria*
Aciaminococcus fermentans DSM20731	1	*Firmicutes, Clostridia*
Acidaminococcus intestini	1	*Firmicutes, Clostridia*
Aquatic bacterium R1-B22	1	*Bacteria*
Aquincola tertiaricarbonis	1	*Betaproteobacteria*
Bacterium E53	1	*Bacteria*
Christensenella minuta	1	*Firmicutes, Clostridia*
Clostidium sp. Usu-S-R3	1	*Firmicutes*
Delftia tsuruhatensis	1	*Betaproteobacteria*
Geobacter grbiciae	1	*Deltaproteobacteria*
Geobacter lovleyi SZ	1	*Deltaproteobacteria*
Sedimentibacter hongkongensis	1	*Firmicutes, Clostridia*
Selenomonas lacticifex	1	*Firmicutes, Negativicutes*
unidentified bacterium ZF3	1	*unidentified bacteria*
Total clone number	41	

Biofilm community structure of the MFC at day 168 (M168B)

Mostly related microorganism	Number of clone	Phylogenetic phylum
Geobacter lovleyi SZ	43	*Deltaproteobacteria*
Geobacter chapelleii	3	*Deltaproteobacteria*
Acidovorax delafieldii	2	*Betaproteobacteria*
Comamonas testosteroni	2	*Betaproteobacteria*
beta proteobacterium SAK19	1	*Betaproteobacteria*
Devosia riboflavina	1	*Alphaproteobacteria*
Geothrix fermentans	2	*Acidobacteria*
Acidaminococcus fermentans DSM 20731	1	*Firmicutes*
Megasphaera paucivorans	1	*Firmicutes*
Megasphaera sueciensis	1	*Firmicutes*
Total clone number	57	
